# “I am alone and isolated”: a qualitative study of experiences of women living with genital fistula in Uganda

**DOI:** 10.1186/s12905-015-0232-z

**Published:** 2015-09-10

**Authors:** Justus Kafunjo Barageine, Jolly Beyeza-Kashesya, Josaphat K. Byamugisha, Nazarius Mbona Tumwesigye, Lars Almroth, Elisabeth Faxelid

**Affiliations:** Department of Obstetrics and Gynaecology, School of Medicine, Makerere University, College of Health Sciences/Mulago National Referral Hospital, Kampala, Uganda; Department of Public Health Sciences, Global Health (IHCAR), Karolinska Institutet, Stockholm, Sweden; Department of epidemiology and biostatistics, School of Public Health, Makerere University College of Health Sciences, Kampala, Uganda

## Abstract

**Background:**

Globally, 2–3 million women are estimated to have a genital fistula, with an annual incidence of 50,000–100,000 women. Affected women remain silent within their communities, and their experiences often go unnoticed. Our objective was to explore the experiences of Ugandan women living with genital fistulas to understand how their lives were affected and how they coped with the condition.

**Methods:**

We conducted 8 focus group discussions (FGDs) with 56 purposively selected women with a genital fistula seeking treatment at Mulago Hospital, Uganda. Data were transcribed and analysed using qualitative content analysis.

**Results:**

Women with a fistula were living a physically changed and challenging life, living socially deprived and isolated, living psychologically stigmatised and depressed, and living marital and sexual lives that were no longer joyful. The women’s experiences were full of life changes and coping strategies, and they used both problem- and emotion-focused coping strategies to deal with the challenges. They devised ways to reduce the smell of urine to reduce the stigma, rejection and isolation. While trying to cope, the women found themselves alone and isolated. Women either isolated themselves or were isolated by society, including by close relatives and their husbands. Their sex lives were no longer enjoyable, and generally, women felt a loss of their marital and sexual rights.

**Conclusion:**

Women with a fistula make adjustments in their lives to cope with the physical, social, psychological and sexual challenges. They use both problem- and emotion-focused coping to minimise their sense of isolation, as well as the rejection and stigma associated with fistula. These findings are essential for counselling patients, families and community members affected by a fistula. In similar contexts, health programmes should go beyond fistula closure and target communities and families to reduce the stigma and isolation faced by women with genital fistula.

## Background

A fistula is an abnormal opening between a woman’s vagina and bladder and/or rectum through which urine and/or faeces continually leak [1]. The global prevalence of fistula is not known but an estimated 2–3 million women are living with a genital fistula, with an additional 50,000–100,000 new cases per year [2–4]. However, this is thought to be an overestimate and the prevalence and incidence may be low [5, 6]. In Uganda, two percent of women 15–49 years old have experienced symptoms of a genital fistula [7]. Most genital fistulas occur after a difficult childbirth or its management and are, hence, termed obstetric fistulas [8, 9]. An obstetric fistula is a public health problem that primarily affects women in low-income countries especially in sub Saharan Africa and Asia [1, 8]. Obstetric fistulas were once common but are now virtually unheard of in Europe and America [4]. Most obstetric fistulas occur after prolonged and neglected obstructed labour [10–12]. In most of these cases, the baby dies during the labour process, and the woman is left with urinary and/or faecal incontinence, having to bear the sadness of the stillbirth and often abandoned by her husband and society [12].

Irrespective of cause, urinary incontinence impacts several aspects of the affected woman’s life, including physical, psychosocial and economic wellbeing [13–15]. Women with incontinence due to a fistula are equally affected and live a stigmatised life with social, economic, psychological, reproductive and sexual repercussions [16, 17], the extent of which varies from one setting to another [17–20]. An evaluation of the UNFPA Uganda Country Programme found that women living with a fistula were either too ashamed to leave their homes, were rejected by their families and community members, or were stopped from working [21]. In Ethiopia, shame, embarrassment and fear of discrimination led to huge efforts by affected women to hide the leakage [22]. The women who were unable to hide the leakage suffered humiliating comments and discriminatory behaviour, sometimes leading to divorce [22]. In Tanzanian, some women with a fistula had separated from their husbands due to their inability to fulfil marital roles and those who were not divorced had to live in separate houses or rooms [19]. The failure to control urine and/or faeces, maintain marriages, bear children or participate in social activities made women with a fistula lose their sense of identity as women, wives, friends and community members [18, 22–25]. Living with a fistula was also associated with the experience of multiple losses, which negatively impacted a woman’s identity and quality of life [19]. In Ethiopia, a study that investigated psychological consequences of fistula among treated and untreated fistula patients showed that women felt cursed by God, were depressed with suicidal ideations, and even when treated they still had social and sexual problems [26]. Women were often unaware of the possibility that, despite having lost their babies during the delivery that led to the fistula, they could become mothers again after a fistula repair [19, 23]. Despite the general trend showing that women with a fistula are rejected, in some cases, relatives are helpful and supportive; furthermore, some women remarry and deliver more children [18, 19, 23, 24].

Few studies have systematically examined the impact and consequences of a fistula on the affected women [25]. Qualitative studies are therefore needed to gain a deeper understanding of how a fistula impacts a woman’s life experience and how women adapt to living with the condition. It is also important to understand how these women maintain their identities as wives, mothers and community members. This knowledge is necessary to inform policy and programmes for the prevention and mobilisation for treatment and rehabilitation of women affected by fistulas. In this paper, we report the findings of a study we conducted in central Uganda. The objective of the study was to explore the experiences of Ugandan women living with genital fistulas to understand how their lives were affected and how they coped with the condition.

### Theoretical framework

Conceptually, the study was informed by the stigma theory of identity management, as described by Goffman [27], and the coping theory, according to Lazarus and Folkman [28].

Goffman defines stigma as “an attribute that is significantly discrediting” [27]. Within the social process, a stigmatised person possesses an “undesirable difference” or “deviance” [27]. Stigma is a constantly changing social process that occurs when five interrelated components converge: namely “labelling”, “stereotyping”, “separation”, “status loss and discrimination” and the playing out of “social and political power” [29]. Discrimination can be individual, structural or self-imposed [29, 30]. Anthropologically, the concept of stigma remains empty and decontextualized if not filled with meaning from people’s lived experiences [31]. Stigmatisation is a pragmatic response to “perceived threats, real dangers, and fear of the unknown” [32]. Stigma can either be enacted or felt: enacted stigma refers to the unfair treatment of others towards the stigmatised person, including discriminatory attitudes and acts of discrimination; whereas, felt stigma refers to the stigmatised person’s internal feelings of shame (self-stigma) and fear of discrimination (perceived stigma) [33].

Coping occurs in response to a stressful situation and is usually initiated by activities or changes aimed at maintaining one’s mental health and emotional wellbeing [34]. Lazarus and Folkman [28] developed a measure called “Ways of Coping”, which consists of predicates, each of which portrays a coping thought or action that people engage in when under stress [34]. Two general types of coping are problem-focused coping and emotion-focused coping [28]. Problem-focused coping is aimed at problem solving or the effort to alter the source of stress, while emotion-focused coping is aimed at reducing or managing the emotional distress associated with the situation. Most stressors elicit both types of coping, but problem-focused coping predominates when people feel that something constructive can be done; emotion-focused coping, on the other hand, predominates when people feel that the stressor is something that can be endured [28, 34].

In this paper, we discuss stigma with regard to the way women perceived their lived experiences, and we attempt to elucidate the central question of interpreting what is at stake for a stigmatised woman living with a fistula in Uganda. The paper also explores the way women coped in their daily lives with this stigmatising condition.

## Methods

### Study design

This was an exploratory qualitative study using focus group discussions (FGDs), a data collection method that enables the researcher to gain a deeper understanding from a group of people about their perceptions, opinions, beliefs, values, understanding and attitudes related to a specific topic [35, 36]. Discussions were held in a setting where participants felt free to talk to each other [37–39]. We utilised group interaction to explore the women’s own and shared experiences [38]. The free interaction among participants generated more information about life with a fistula, as the participants were able to build on each other’s responses [36, 40].

### Study setting

Data were collected from October 2012 to March 2013 at Mulago National Referral and Teaching Hospital, located in Kampala, the capital city of Uganda. The hospital provides routine female genital fistula care, including prevention, treatment and rehabilitation. Annually, the centre receives over 300 fistula patients. The patients are referred from all over the country and receive free treatment. The patients are screened at the outpatient fistula clinic by fistula surgeons and once a fistula is confirmed, they are admitted on the urogynaecology ward. Upon admission, the patients are counselled about the surgery and possible outcomes and are given instructions about what to do after surgery both in hospital and after discharge. Patients then undergo surgery in a dedicated fistula theatre. Following surgery, they stay on the ward for 14 days under the care of both specialised fistula nurses and surgeons. It is during this time, from admission to discharge, that we were able to conduct the FGDs.

### Study participants

To ensure maximum variation in the group, participants were purposively selected from both women awaiting surgery and those already operated on. We included women confirmed by a specialist as having or having had a genital fistula, irrespective of whether they had already undergone surgery or not. The composition of the groups was determined a priori based on age, marital status, whether or not they had children, fistula repair history, and length of time they had lived with a fistula to maximise information richness. A total of 56 women, aged 14 to 60 years of age, participated in the eight focus group discussions with a group composition of five to ten women (Table 1).

**Table 1:** Characteristics of respondents in the focus group discussions

FGD	Group composition	Age	Women in each FGD	Duration of leakage
1	Single, young, all got still births	14–20	10	5 months −3 years
2	Divorced and have children	26–55	10	5–20 years
3	Divorced and having no child	26–45	9	5–25 years
4	Divorced previous failed repair	19–35	5	2–20 years
5	Married previous failed repair	26–41	5	3–27 years
6	Mixed (3 married and 3 divorced), no previous repair	22–33	6	1.5 month- 4 years
7	Divorced, lived long with fistula, no previous repair	38–60	5	7–40 years
8	Married, young, no previous repair	16–20	6	3 months-5 years

### Data collection

The first author, together with a female social scientist with experience in collecting qualitative data using FGDs, conducted the discussions. The social scientist received basic knowledge with regard to fistula. Nurse/midwives at the fistula treatment centre conducted the initial identification of the participants following confirmation that they had a fistula. During each discussion, a midwife acted as an observer. The first author initially observed and later moderated some of the discussions. A FGD guide (Table 2) developed by a multidisciplinary research team comprised of a fistula surgeon, gynaecologists, a midwife, a social scientist and public health specialists was used. The guide had open-ended questions. The FGDs took place in a quiet, private room on the urogynaecology ward. The venue was organised with a comfortable table, which enabled the women and the moderators to sit around facing one another, with an audio recorder in the middle of the table. All discussions were audio recorded and lasted between 45 min to 1½ hours. The discussions were conducted in Luganda, a local language understood by both the participants and moderators. Probing was done for shared or conflicting values between the participants and to get to the rich information on the issues discussed. Though we had a pre-defined group selection criteria to help ensure homogeneity in the groups and information-rich discussions, the number of discussions was not pre-determined. We stopped conducting further FGDs when we reached information saturation [38], a point at which we were no longer getting any new experiences from the women, for all the information being gained appeared to be repeating information already provided by the earlier groups.

**Table 2:** Focus Group Discussion (FGD) guide: Life with a Genital Fistula: Experiences among Women Seeking Treatment in Mulago Hospital, Uganda

*A.*	Introduction/consent
	The discussion begins by introduction, giving information about the study to respondents and seeking consent for participation and recording voices.
*B.*	Questions to guide the discussion: Use probes to ensure clarity
1	What are some of the reasons (causes) why women in this community may develop leakage of urine/faeces?
2	Can you give an account of life with a fistula from the time a woman in this community realizes that she is leaking urine and or faeces up to when she finally decides to seek medical help?
3	In what ways does leakage of urine affect a woman’s relationship with other people including her relatives, neighbours, peers and spouse when she has a fistula?
4	How does leakage of urine impact on a woman’s sexual and reproductive life in this community?
5	How does leakage of urine following fistula affect the marital rights and responsibilities that a woman should otherwise enjoy?
6	Now let’s talk about seeking medical care for women with fistula. Can you tell us how it is in this community? Kindly tell us if there are any difficulties and the measures women use to overcome the difficulties?
7	How does a woman feel or would feel if this hole is repaired successfully. How does repair influence her social, sexual, and psychological well being?
8	What message do you have for the pregnant women? Suggest what they can do to avoid this problem of fistula following delivery?

### Data management and analysis

Data from all the FGDs were immediately stored on a computer and transcribed and translated into English from the local language. The same social scientist that moderated the discussions translated the discussions from Luganda to English. The first author, who is fluent in the local language, listened to the audio interviews and, together with the translator, checked for consistency and a proper interpretation of the discussions against the transcripts. The transcripts were then analysed using a qualitative content analysis [41], through which both the manifest and latent messages were brought out. The field team, together with the first, second and last authors, read through the transcripts and notes of the discussions several times, identifying meaning units. The meaning units were then condensed, coded and put into categories, from which themes emerged. We used open-code software [42] blended with a manual analysis. Open code helped to organise the transcripts into a format to analyse, identifying meaning units, assign codes, categories and themes. Table 3 illustrates the process of analysis from codes to categories and themes. Though we had eight questions on the discussion guide (Table 2), in this paper, we have concentrated on mainly four questions (2,3,4,5,) to allow the women to describe the ordeal of life with a fistula. The results from the rest of the questions (1,6,7,8) focus on cause, treatment and prevention of a fistula and a different paper is being prepared. The unit of analysis was a focus group discussion.

**Table 3:** Living with genital fistula- illustration of codes, categories and themes

Codes	Categories	Themes
Waking up from sleep… urine has spread to all the bed up to the top, to the extent of wetting the hair.	Too much urine: Occupied with avoiding wet outer clothes	Living a physically changed and challenging life
I wash daily like a ‘Nakawere’ (woman with a new-born)		
I use many pieces of cloth as pads.		
Wounds do not allow me to walk properly. I walk like a lame woman	Burnt by strong urine or pad: living with genital sores and rashes	
The pads burn my private parts and I am full of wounds due to padding myself all day long.		
Urine that comes when you have not drunk has a very bad smell	Urine too smelly to bear	
Use herbs for wounds and smell but did not work		
Friends changed, they are not like how they used to be.	Socially Rejected	Living in social deprivation and isolation
My siblings shunned me and could not even enter my bedroom		
They don’t want to get close to me thinking that I am dirty. This makes those of us with the fistula condition feel very bad		
Ashamed of visiting friends		
I quietly suffered	Socially Isolated	
I cannot attend to visitors due to smell		
When others are beginning to be happy, the urine starts dribbling		
Other people do not want to come near me, they say I smell bad		
Neighbours feel bad when I feed their children, I therefore, keep away		
My father used to verbally abuse me (… die like a dog, you deserved the problem, you are a disgrace to family).		
I also don’t go to their places because I feel like a burden		
Whoever gets to know of your condition, he/she can’t accept you to work		
She is like a child, she urinates wherever she goes	Perceived stigma (from friends and relatives)	Living Psychologically stigmatized and Depressed
You even fear to hang it (padding cloth) for drying		
Fistula feared contagious, wherever I sit nobody else would sit there.		
I saw my mother getting irritated by my padding cloth		
They mock me, spit at me, laugh at me, and do not talk to me.		
They even stopped their children to come closer to me.		
They stop their children from coming to my place and also playing from there in order to stop me from giving them edibles		
People around do hate me.	Enacted stigma (from self)	
Everybody makes my life difficult.		
I hated myself and reached an extent of wanting to kill myself	Depressed	
Fear wetting self in front of other people. Feel misery and have no peace		
I am widened, feel pain in the vagina, and different from other women	Changed body	A marital and sexual life no longer joyful
He left me and got married to another woman	Abandoned by husband	
I lost my marriage		
He (husband) told me that he can’t stay with me		
Too much cleaning to have sex	Sex no longer normal	
I am never in mood for sex.		
So this reduces my appetite for sex		
He does not enjoy my company (sexual intimacy)	Sexually denied and rights violated	
Living under one roof but different bed/room		
Not producing again		
He forced me to have sex, actually he raped me		

### Ethical considerations

We received ethical approval for this study from Makerere University College of Health Sciences, School of Medicine Research and Ethics Committee (#REC REF 2011–104), Uganda National Council for Science and Technology (UNCST) (HS 1337) and the Regional Research Ethics Committee in Stockholm (No.2012/474-31/2). Participants were provided with both oral and written information about the purpose of the study, the data collection procedure, confidentiality and voluntary participation, including the right to withdraw from the study at any time without denial of treatment. The study participants gave oral consent and also signed or put the right thumbprint on the group consent form. Four participants under 18 years of age having been previously married, conceived and given birth, consented as emancipated minors, which is in agreement with Ugandan policy and ethical guidelines [43, 44]. Though initially we planned to exclude minors, we included them on recommendation by Institutional review boards at Makerere and UNCST since age is a known risk factor for fistula. Excluding the experience of these teenage women would mean leaving out experiences from a very important group. In the transcripts, the participants were represented by pseudo-initials to protect their confidentiality. The participants were given between 5,000 and 20,000 Ugandan Shillings (USD$ 2–8) to refund transportation costs (depending on the participant’s distance from the study site) and/or as compensation for the time spent in the discussions.

## Results

The results are presented below in two sections: the socio-demographic characteristics and the women’s experiences of living with a genital fistula. Women’s experiences are presented according to the four themes that emerged, and we use relevant quotations to illustrate and validate the categories.

### Socio-demographic characteristics

A general profile of all the study participants is presented in Table 1. Altogether, 56 participants between 14 and 60 years old participated in the eight FGDs. The median age for the participants was 26 years with an inter-quartile range of 19 to 38 years. The women had lived with a fistula for a period ranging from one and a half months to 40 years. A total of 32 women (57 %) were divorced/separated, 14 women (25 %) were still married and 10 women (18 %) got the fistula when single and were still not yet married. Of the 56 women, one had a fistula from childhood (congenital ectopic ureter) and the remaining had a fistula following childbirth (obstetric fistula). The group selection was important for the women to freely discuss their experiences. The women generally gave a coherent story regarding their experience with a fistula, irrespective of which group they belonged to; and hence our results are not segregated per group, as the study was not designed to compare intergroup variations.

### Experiences of living with a genital fistula

In the analysis of the lived experiences among women with a fistula, four themes emerged (Table 3). The four themes were: living a physically changed and challenging life, living in social deprivation and isolation, living psychologically stigmatised and depressed and living a marital and sexual life that is no longer joyful. The four themes were inter-related and occasionally overlapping from the way the women described their experiences. Generally, the women’s experiences were full of life changes and strategies to cope. The coping responses cut across all four themes and are hence not presented separately.

#### Living a physically changed and challenging life

The women’s lives generally changed the moment they realised they were leaking urine uncontrollably. They were wet all the time, a challenge they had to cope with by devising ways of passing less urine and avoiding being noticed by those around them. In response to the leakage, women in all FGDs decided to drink less to reduce being constantly wet. However, drinking less made them produce concentrated and smelly urine, which then caused “burns” and sores on the genitals and thighs. All the women padded themselves all the time using locally devised pads that would eventually “burn” them. All the time, the women were pre-occupied with ways to reduce being noticed when they were wet, which was physically challenging as demonstrated in the following quote:

*Waking up from sleep, you find urine has spread all over the bed up to the top, to the extent of wetting the hair. Then you feel the burden of changing the beddings to place the dry ones…you fear to drink so that urine does not come much. Then again the urine that comes when you have not taken anything has a very bad smell.***FGD 6**

Women reported the dire situation of living with constant leakage and, as a consequence, with an offensive urine smell. Physically, the women themselves experienced the bad smell of strong urine and often felt ashamed. However, they also attributed the response from others to the physical effects of urine smell as they always felt like outcasts in the company of other people

*Where I pass, they say, “there she is; smelling”. Both the young and the old share the same message and keep saying that I smell. Everybody makes my life difficult. I cannot go to church or visit friends or attend to visitors due to the smell… and I fear that I will wet myself in front of people.***FGD 3**

The physical effects of the bad smell were even worse for those who leaked faeces in addition to urine. They felt the situation of bad smell would drive away anybody they encountered. They often took a much time cleaning themselves to reduce on the smell. One woman who leaked faeces narrated her ordeal in delaying her husband every morning, as she would be in the toilet cleaning herself.

*I made it a habit to visit the toilet every morning; if I don’t go there, faeces come and I smell bad. I quietly suffered … he [husband] would wait for me every morning when am just in the toilet cleaning myself. Whenever he would ask, I would tell him to go and leave me to come later and he did not know why.****FGD 5***

It was difficult indeed to conceal the leakage, causing women to change the way they padded themselves. They devised a locally made special pad, which has many layers of cloth plus a plastic sheet to contain the continuous leakage of urine. The participants also had to frequently wash their clothing to avoid the urine smell and the rash around the genitalia and thighs.

*The most difficult situation I have passed through is washing; I wash every day like a Nakawere (*woman who has just given birth*). You have to keep washing to avoid smelling. You can’t put on the same cloth twice. Secondly is the rash I get down there as a result of heat and urine.***FGD 7**

The participants faced the dilemma between wanting to drink much water to reduce the smell but risking massive leakage, and drinking less water to reduce the leakage but risking the increased smell. The continuous leakage of urine, the padding and the concentrated urine burnt their thighs and private parts. This situation made it increasingly difficult to pad themselves because of the burns, and the resulting burns (wounds) often limited their body movements.

*You fear to drink because you are going somewhere so that urine does not become much. Then again, the urine that comes out when you have not taken any thing, the smell is very bad.***FGD 6**

*I have been with the condition for two years and life has been very difficult. I use pieces of cloth as pads. The pads burn my private parts and I am full of wounds due to padding myself all day long. These wounds do not allow me to walk properly. I walk like a lame woman.****FGD 1***

To mitigate the physical effects of leakage and the smell, the women had to look for local remedies. Some women used herbal medication to treat the burns. Some reported that herbs also helped to reduce the leakage of urine. However, most women reported they did not achieve the anticipated benefits and that they continued leaking.

*My husband put in a lot of effort in consulting from older women; they told him about some herbal medicine I could use. One woman gave him herbs that I was supposed to sit in and I did. The other herbs were for putting in tea. I had some relief. After 6 months, the leaking lessened*. ***FGD 7***

*I tried herbal medicine but it failed to heal me. They used to mix the herbs in a small, 5-l plastic container and you take. They were sour but because you want to be ok you continue taking… but the herbs never stopped the leakage.****FGD 4***

#### Living in social deprivation and isolation

All the women in the different group discussions felt they were socially isolated. They shared the view that because of their condition, they would either isolate themselves or be isolated by relatives and friends. They attributed the social isolation to the rejection they received from friends, neighbours and relatives as a result of the leakage and the smell, and isolation was a coping mechanism for most of them.

*… my friends and my relatives are irritated and they don’t want to come to my place anymore. I also don’t go to their places because I feel like a burden to them. We both fear each other. The neighbours feel bad when I give food to their children; as a result, they stop their children from coming to my place or playing from there.***FGD 7**

*I do not have friends anymore; even members of my family do not want to associate with me. I cannot talk to anybody for they are pushed off by the smell of urine. I am alone and isolated.****FGD 1****.*

These women were socially excluded, as they could not perform what they perceived as their culturally normal duties, like preparing food. They could not touch anything, for they were considered unclean, and if they did, whatever they touched would be thrown away. This made the women with fistulas feel socially rejected.

*Nobody wants to associate with you because of smell. I was denied of everything. I could neither touch on anything nor prepare food for the family. Whenever I prepared food or touched on a cup or plate my senga (paternal aunt) would not eat or take that tea.***FGD1***The neighbours feel bad when I give food to their children; as a result, they stop their children from coming to my place or playing from there.***FGD 7**

Participants shared stories of being rejected and abandoned by relatives upon realising that they were continuously leaking urine. This rejection was across all age groups. Children and close relatives laughed and mocked the women and this worsened the rejection and prompted the women to isolate themselves as a form coping.

*Children who do not understand your condition, laugh and sneer at you, they point fingers saying that “look at that woman; she is like a young child; urinating wherever she goes”.***FGD 7**

The women often concealed the fact that they were leaking urine. Women in all groups generally agreed that it helped if they never disclosed their illness to relatives and neighbours. The women said that the rejection worsened as time went by, as initially, the husband hoped the woman would heal. However, majority of participants reported that their husbands abandoned them after realizing that the wife’s condition might not heal.

*…At first, I didn’t tell him what was going on with me; I hid it from him for about 3 months. Whenever I would leave our bed and he realizes that it’s wet, he would ask me if I had urinated on the bed, I later told him I had got the problem during childbirth. As soon as I admitted the truth to him, he left me in the house alone without help and got married to another woman… so, when my relatives saw that I may die in the house, they came, picked me and brought me to hospital.****FGD 7***

The experience of being ostracised by relatives, friends and the community was, according to the participants, too much to bear. The majority of participants reported ridicule, scorn, verbal abuse and non-verbal mockery *(kwenyinyala)*. What seemed to hurt most was the abuse from relatives and parents who voiced that the women with a fistula were a disgrace and deserved to suffer from a fistula.

*… my father used to verbally abuse me. Whenever he got drunk, he would start abusing me that am going to die like a dog. He would say that I deserved the problem [fistula] and would command me to leave his house/home saying, “You are a disgrace to this family”.****FGD 1***

Various reasons were given for the choice of self-Isolation among women with a fistula. The women chose to isolate themselves from friends, as they could not afford to sit for a long time and chat and, hence, decided to abandon their former peers and friends.

*Friends changed! They are not like how they used to be. For those whom I used to sit and chat with for a long time, it can’t happen anymore. This is because; if she overstays, you will leave her there and you hide yourself to change pads and from there, she will say you no longer love her. Therefore, I just hide my problem from most of them and I decided to chuck most of them.****FGD 6***

The other reason given by the women to isolate themselves from relatives was because relatives had spoken ill of them, and they could not continue with such relationships.

*When my sister in law visited me in hospital, she found out that I was leaking. She spread the news about my condition that I was leaking urine. I decided to remain alone and cut off all of them. I do not invite them nor visit them so am at home with no friends.****FGD 4***

Women with a fistula shared that they had lost friends, because no friend would tolerate their toilet habits and thought their friends would feel ashamed to be in their company when in public.

*Those who used to go out with me have cut off the relationship because of the condition. When I go out with a friend, and she notices that I go out to the toilet all the time, she changes her minds and the next step is to drop you by giving excuses. They don’t want to go with you because they feel ashamed to be with you in public.****FGD 6***

Leaking urine affected the women’s social interactions so much that they preferred to stay alone as a coping response. The women could not participate in activities they were culturally supposed to enjoy, such as entertainment and cooking for visitors for they feared wetting themselves as they participated.

*Ever since I got the problem, I felt so bad. All the time you are in fear of not wetting yourself in front of others. You are invited for functions or parties but you cannot attend due to fear and also changing the pads all the time. I became very shy and feared hanging around with my people. Even when I got visitors, I felt I could not prepare them food because I was afraid of their attitudes towards my condition and me. I leave a pool wherever I sit. I therefore avoid gatherings.***FGD 5**

Another coping response involved avoiding public functions and meetings. Some participants explained that they avoided public functions such as funerals and attending church because of the leakage and smell of urine. Those who attended social and religious functions had to develop some behaviour modifications for example, women avoided standing up or raising their voices when singing.

*Life has been very difficult for me. I hate myself and cannot mix with people. I cannot attend any party, social function or even go to church. If I am to go anywhere I have to tie bundles of clothes around me and when they get wet they burn my thighs and private parts. If I am at church and praising, I don’t dance and jump because if I do, a lot of urine and faeces will come out. I even sing at a low tone because of the same reason. I would never enjoy the service or anything for I would be preoccupied with how to avoid my outer clothes being wet.****FGD 3***

Some women demonstrated that urine leakage affected their socioeconomic activities Women affected by a fistula were unable to continue with income-generating activities to support themselves. The main reason stated was that they felt they unprepared to continue working because of pre-occupation with how to cope with effects of leakage. However further showed that social interaction was also at play, as the women felt they would be mocked at work. Those who had a small retail business feared that nobody would buy from them if they tried to take their goods to the market. Additionally, all women felt that no one was willing to employ a woman leaking urine, as demonstrated in the following quotes:

*This condition has hindered us from doing our usual work that could give us income to support our selves. …I no longer take my things to the market because I fear people will not buy from me.****FGD3***

*There is no way how you can hide this problem because it’s a complicated thing to hide. So whoever gets to know of your condition, he/she can’t accept you to work.****FGD 6***

However, some participants reported having sympathetic relatives who even helped out during this illness and who advised them to seek care.

*… after knowing the condition, they [relatives] felt sorry. They even contributed to the fee that was needed to treat me when I went to the hospital. It was very expensive…they mind a lot about my life because whenever they hear any announcement about fistula treatment, they call to inform me.***FGD 4**

#### Living psychologically stigmatised and depressed

Though it was difficult to have a sharp divide between social and psychological effects of living with a fistula, women with a fistula demonstrated actions that showed they were stigmatised and often depressed by the condition of leaking urine and or stool. Almost all the participants reported having faced some form of stigma. The stigma manifested itself as feelings that others were irritated by the smell and leakage of urine. The women were constantly worried that others would notice their problems. Stigma was also experienced through the behaviours of others, especially close relatives, towards women with a fistula.

*They don’t want to get close to me thinking that I am dirty. So I don’t feel like going to anyone’s place to visit so as not to be an object of contempt. Even when you are at someone’s place and you wash your padding clothes, you even fear to hang it for drying. One day, I saw my mother getting irritated about my padding cloth.***FGD 7**

Women with a fistula lived in a state of distress and fear from the behaviour of relatives and neighbours. The physical effects of leaking urine, the bad smell and sores together with the fear of being noticed in public forced the women to live in a state of psychological distress. The women themselves felt it was not worth being in public. On the other hand, relatives and neighbours distanced themselves from women with a fistula because of the perceived fear that the condition might be contagious.

*What I have experienced with my neighbours is that they fear that I could spread the condition to them. Some of them think that it is contagious so where you have sat, no one would want to sit there, especially in my case who stays in a rented room. Neighbours don’t even want me to sit on their verandas and they are also cautious and scared of sitting at my place. Even after pouring water you have used outside, no one would want to get in contact with it or jump over it. This makes those of us with the fistula condition feel very bad.***FGD 7**

The stress of living with a fistula was compounded by worries that there may be no cure for the condition. Furthermore, others confirmed the participants’ fears by saying the condition was for life. As a result, some contemplated ending their lives. Most of the women had ever developed suicidal ideations and felt their life was not worthwhile.

*…People were saying that the condition could never be treated, so I will have to die with it. I started fearing to live with the problem the rest of my life. They said that I would never be o.k. So if am wise, I should just get a rope to hang and kill myself! I was going to do it but my mother stopped me.****FGD 8***

#### A marital and sexual life that is no longer joyful

Most of the women felt their marital and sexual lives were no longer joyful. Others felt they had lost their marital and sexual rights altogether. Their bodies had changed, and they were no longer like other women, because they missed the happiness they formerly enjoyed. Their sex lives had changed. Many described their sex lives to have been negatively impacted because of the excess fluid, which was detestable to both the women and their partners. All the participants agreed that sex was a major challenge. It was no longer as it used to be, and there were many difficulties to overcome when having sex.

*When it reaches time to play sex, he tells you do this, and you have to first put there the cloth to dry urine, then you lay a polythene cover and by the time you start, urine is much again. This makes him lose the erection and a feeling comes that may be its urine that made him lose the erection. He just forces himself to do it but deep inside, he is not enjoying and even myself, I do not enjoy due to fear that when you increase on the speed, urine will come in large quantity so you do it slowly. In fact, you do not make him happy like how you used to do before the problem.****FGD 5***

Participants described the feeling of a changed vagina. Some participants reported that their partners felt that sex was different now compared to the period before the development of the fistula. In addition, some partners told their wives that sex with them was different from sex with women without a fistula. Furthermore, participants felt physically widened, and as a consequence, their appetite for sex had decreased.

*I hate myself because, when am cleaning myself, … I feel I widened … I feel pain in the vagina so this reduces my appetite for sex. When I squat to urinate, it becomes worse and air just blows into me. Even the man I got, at first he did not know but afterwards, he came and asked me, what is the problem? As you are different [sexually] from other women!****FGD 8***

Some partners were unable to tolerate having a wife with a fistula and separated from their wives. Alternately, those couples that stayed together often had separate beds and only came together for sex.

*… My first husband chased me away and I got myself another one I am having today, but we have separate beds. I feel bad to see that I do not feel the warmth of a man and the man also does not feel mine.***FGD 5**

Participants said their right to sex was denied, because their partners, as a strategy to cope with the changed sexual experience, looked for other “normal” women and left their wives with fistulas sexually starved. Even when the women tried other men, the fluid was too much and unpleasant, and the men did not return.

*Yes, he continued with sex, but he was unstable. He preferred going to his “normal” wife than being with me who leaked a lot of fluid during intercourse. I got a few other friends but the intercourse was still messy due to the release of a lot of fluids and they moved on.***FGD 7**

On the other hand, some participants were forced into sex even when they had pain, a situation that they interpreted as rape.

*When he is badly off he insists on having sex and after forcing you, you get wounds all over the place. In fact, penetration is painful. It’s actually rape because; there is no feeling at all but for him, he insists on having it done*. **FGD 6**

The few participants who still wanted to have sex were challenged by the fact that husbands divorced them to marry other wives. Women reported that in this setting, men are culturally the initiators of sex. Therefore, the women felt that being divorced and yet being unable to initiate new sexual relationships was a violation of their sexual rights. The women thought a fistula had stood in their way, even when they still had a desire for sex.

*Our rights were violated. We don’t play sex; not because we lack feelings; … this condition [fistula] made our husbands- the custodians of sex, leave us for other women. Men no longer want to socialize with us and ask for love [sex] because of our condition …yet sometimes we have feelings for sex.***FGD 2**

Most of the participants had completely abstained from sex. Others assumed that almost all women with a fistula were not having sex. The participants feared being talked about if sex did not go right and, hence, decided to avoid it altogether.

*… I get men who come and request me for marriage, while others want sex, but I know my problem. You fear that you might play sex with that man and he goes out and talks about you, so I decided to leave everything.****FGD 4***

Most participants decided to continue life without sex after facing sexual challenges. Others often stayed away from their partners to avoid abuse and neglect. Separation or divorce was very common among the participants. Most reported that either they left their abusive partners or their partners just abandoned them or sent them away.

*I feel I cannot be loved again. …In my mind I ask myself who will come to me and say that I love you when I am in such a condition. I lost hope of getting another partner in life.***FGD 1**

## Discussion

Our findings show that the lives of women with a fistula are full of challenges and adjustments. These women were physically challenged, felt rejected, isolated and stigmatised against, and their marital and sexual desires had been greatly affected. Based on the social stigma theory [27], both enacted and perceived (felt) stigma, are weaved into the lived experiences of women with a fistula, making them feel alone and isolated. In a bid to maintain mental and emotional wellbeing, the women devised ways to cope with the challenge of leaking urine, but some of the coping strategies eventually turned into stressors that would lead to even greater stigma for the women. Their lives revolved around ways to prevent the leaking of urine or faeces from being noticed, to reduce the smell and to maintain their relationships with their husbands, relatives and the community. Despite all their life adjustments to cope with this challenging morbidity, the women still found themselves lonely and isolated..

All the four themes against which the findings are presented were interlinked and punctuated with strategies to cope. The findings indicate that women perceive leaking urine to be a discrediting situation, and they consider themselves to be different from other women; thus, they separate themselves from the rest of society. The findings echo what was previously found in other low-income settings in Africa and Asia, with the general agreement that a fistula has negative social and psychological implications that result from its physical manifestations [16, 20, 22, 23, 26, 45–50]. Drawing from experiences of women with incontinence irrespective of cause, our findings show urinary incontinence following fistula impacts on several aspects of the affected woman’s life including physical, psychosocial and economic wellbeing [13–15]. In addition our findings show that the worst suffering may not be from physical manifestations of the fistula, but rather from the social, psychological and sexual impact [16].

Our findings show there is a series of events that takes place in the life of a woman with fistula beginning with the physical effects and ramifying to social, psychological and sexual challenges due to the leakage and smell of urine. Upon developing a fistula, a woman’s life changes suddenly with the onset of symptoms, which requires an immediate lifestyle change to cope with the leakage, such as padding and drinking less, as well as isolating and cleaning herself frequently, among other lifestyle changes. The women drink less to reduce the amount of urine leakage and in a bid to avoid notice when outer clothes become wet. However, the less they drink, the more the urine becomes concentrated. Eventually, they acquire “burns” from concentrated urine and from the improvised pads used to avoid being noticed that they are wet. The strong urine creates an offensive smell, which may directly push away relatives and friends, or the women may decide to hide and isolate themselves before they are noticed. The women perceive the smell of urine to be a threat and, thus, develop feelings of shame (self-stigma) and a fear of discrimination (perceived stigma) [29, 30, 33]. This marks the beginning of social isolation and rejection from the community. These findings in the Ugandan setting compare with findings in other countries in Africa, where the experiences of women with a fistula have been studied [16, 20, 22, 23, 26, 46–50]. In Tanzania, Mselle et al. found that women living with a fistula experience a deep loss of body control, loss of the social role of being a woman and a wife, loss of integration in the social life and loss of dignity and self-worth [19]. A study from Ebonyi State, Nigeria, similarly found that women endure physical problems including sores and blisters due to constant wetness and friction, which affects their ability to participate in daily chores as women [48]. Engender Health and the Women’s Dignity Project in Tanzania also found that women experience social, psychological and economic problems following the physical manifestations of fistulas [51].

The gender expectations of being an African woman in general, and a Ugandan in particular, ceased to hold for the women in our study [52]. These roles that span from the physical, social and sexual lives of the women changed for women affected by fistulas. The women stopped having sex and if they had sex it was not enjoyable. Additionally, most of the women were divorced and have no living child, which made them lose their dignity as wives and mothers. While in some cases, the community isolated the women with a fistula, in other cases; women actually decide to isolate themselves to avoid abuse, ridicule and perceived stigma. Generally, women experience a loss of womanhood and its associated roles. This was evident not only with regard to sexual life with their husbands, but also with regard to other social responsibilities, such as cooking, hosting guests and participating in social functions. The woman’s social position was lost, and she was left alone and isolated. Our findings compare with what has been reported from other African studies [47, 48, 50, 53]. Reports from Nigeria, Kenya, Ethiopia and Ghana show that women with a fistula live socially isolated lives, prohibited from participating in communal activities, which leaves the women isolated and psychologically tortured, as they cannot even eat or drink with their husbands and relatives [47, 48, 50, 53].

Our findings further show that having a fistula is a typically stressful and challenging condition in which individual women try everything they can to cope. From the coping theory according to Lazarus and Folkman [28], both problem- and emotion-focused coping are exhibited by women with fistula. In particular, the women who adopt problem-focused coping are proactive about their condition and future. They tried to clean themselves regularly, for example. Some chose to simply keep quiet. Others tried local herbs from traditional healers, hoping the condition would go away. Compared to other studies, it is clear that women with a fistula choose coping strategies depending on their local setting, though the general trend is they will simply search for what will make their social outlook improve. For example, in Nigeria, women had to bathe regularly, use old wrappers to form pads and use perfumes and powder to cope with the associated urine smell [48]. Other studies, which have examined the coping responses of women with a fistula, have also found that women with a fistula tend to maintain hygiene and cleanliness and are constantly padded [16, 20, 51]. Similar to what has been reported in other [45, 51, 54], our findings show that women with a fistula drink and eat less, use various herbal remedies and tend to be isolated. Alternately, our study showed that women who adopted emotion-focused coping strategies felt shame and tended to avoid others, including peers, relatives and community members. The women lived in a state of self-enacted stigma, isolation and rejection. These women preferred isolation, did not attend social and religious functions and felt it was not worth continuing to have sexual relations with their spouses. These findings compare with what has been reported in other studies from the rest of Africa, especially in Ethiopia, Tanzania and Nigeria [16, 45]. The women who are stigmatised against live on the margins of society, as a result of their isolation because of the smell, embarrassment and fear of ostracism from the community [16, 17, 20, 54]

From the stigma theory, to be stigmatized there must be an attribute that is significantly discrediting to be stigmatised against [27]. The stigmatising nature of living with a fistula was discrediting and these women could not engage in gainful employment, as they believed that nobody would employ them because of their condition. This means they have to depend on others who can provide support and help meet the financial requirements that come with this condition. These findings agree with what other studies have reported in that women with fistulas have no gainful economic activities and are hence, financially dependent on others [17, 55]. The women in this study, like women in other settings, were perceived as unclean and had to cope with pain, discomfort, shame, depression, isolation and stigma from the community, as well as from their own spouses and families [17, 54].

The women with fistula in our study faced physical challenges, stigma and isolation, as well as the problematic relationships with spouses and the community at large. The damage extends beyond the physical hole in the bladder and the leakage; rather, it involves a complex interaction with the environment a process that often leaves them socially isolated. In Tanzania, women were still discriminated against, even after they had received treatment [54], while in Nigeria, the treated women had to demand a certificate of good health to serve as proof they have been fully treated and can thus be allowed to participate fully in community affairs [48]. We, therefore, need to have community programs that will focus on reintegration by using lessons learnt from the life experiences of the women affected.

### Methodological considerations

We acknowledge that there are methodological limitations that may impact the findings. The qualitative design was chosen because we wanted to gain a deeper understanding of the experiences of the women living with fistulas. The study participants were women seeking care, and thus, this may be representing a group who receive support from relatives; further, we may have missed the very needy women hiding in the villages. We acknowledge as a limitation the fact that we did not include in-depth interviews to complement FGD. This triangulation during data collection could have further enriched our findings on women experiences that were not brought out in the FGDs. Audio recordings, verbatim transcriptions and the direct quotes from respondents improve credibility, conformability and dependability. We believe the experiences of the women in this study are relevant to other settings with similar socio-economic contexts, especially in low-income countries in Africa and Asia, indicating transferability. The research team was multi-disciplinary at all stages, including the study design, data collection, analysis and manuscript writing, with researchers experienced in using qualitative research methods. This team composition, together with the careful thick description of the research process and analysis, enhances the trustworthiness of our study. The fact that the fistula surgeon was involved in the discussion process could influence our findings but using an independent social scientist to moderate most of the FGDs minimised this bias.

## Conclusions 

The findings from this study show that women with a genital fistula live a challenging life and face ostracism. The women live a life of physical, socioeconomic, psychological and sexual challenges. Unlike other qualitative studies on the experiences of women with fistula our findings give a detailed account of the sexual challenges women face. They are socially isolated and rejected, stigmatised and depressed and there is no joy in their sexual life. In attempt to fight the stress that results from leakage, the women with fistula adapt both problem- and emotion-focused coping strategies. Emotion-focused coping is mainly applied through avoidance coping, such as building on the life adjustments the women make in their physical, psychosocial and sexual lives. The results of this study are important for those countries in Africa and Asia, where fistula prevalence is still high. Regarding transferability, the experiences of the women in this study are relevant to other settings with similar socio-economic contexts, especially in low-income countries in Africa and Asia. The fact that these women’s stories are characterised by community isolation and stigma calls for the careful design of community programmes to target the grassroots with messages that may help identify the women for treatment and also reintegration.

The findings can be used as a resource for health education and advocacy programmes aimed at reducing stigma and increasing social support for women and girls with genital fistulas. Further research is recommended however, to explore the experiences of women following treatment and to establish the community needs for the women when they are discharged from hospital after treatment. In the Ugandan setting it is not known how women adjust to life after treatment, or whether they remain stigmatised following treatment. Regardless of the need for further research, our findings may aid policy and public health programmes to go beyond surgery for closure of the fistula and to involve communities and families in targeted health education, as these women suffer from more than just the leakage.

## Abbreviations

FGDs: Focus Group Discussions
UNFPA: United Nations Fund for Population Activities

## References

[1] Lewis G, De Bernis L (2006). Obstetric fistula: Guiding principles for clinical management and programme development.

[2] Murray C, Lopez A. Health Dimensions of Sex and Reproduction. Cambridge, Massachusetts, USA: Harvard University Press; 1998

[3] Tunçalp Ö, Tripathi V, Landry E, Stantonc CK, Ahmedc S (2015). Measuring the incidence and prevalence of obstetric fistula: approaches, needs and recommendations. Bull World Health Organ.

[4] Wall LL (2006). Obstetric vesicovaginal fistula as an international public-health problem. Lancet.

[5] Adler AJ, Ronsmans C, Calvert C, Filippi V (2013). Estimating the prevalence of obstetric fistula: a systematic review and meta-analysis. BMC Pregnancy Childbirth.

[6] Ahmed S, Tunçalp Ö (2015). Burden of obstetric fistula: from measurement to action. Lancet Glob Health.

[7] Uganda Bureau of Statistics (UBOS), ICF International Inc, UBOS and Calverton (2012). Uganda Demographic and Health Survey 2011. Kampala, Uganda.

[8] Partners FIGO (2011). Global Competency-Based Fistula Surgery Trainining Manual.

[9] Hancock B (2005). First Steps In Vesico-Vaginal Fistula Repair.

[10] Waaldijk K. Obstetrics fistula surgery art and science: Edinburgh UK: Campion press; 2008.

[11] Hancock B, Browning A (2009). A text book of practical obstetric fistula surgery.

[12] Wall LL, Arrowsmith SD, Briggs ND, Browning A, Lassey A (2005). The Obstetric Vesicovaginal Fistula in the Developing World. Obstet Gynecol Surv.

[13] Hayder D, Schnepp W (2010). Experiencing and managing urinary incontinence: a qualitative study. West J Nurs Res.

[14] Lagro-Janssen T, Smits A, Van Weel C (1992). Urinary incontinence in women and the effects on their lives. Scand J Prim Health Care.

[15] Subak LL, Brown JS, Kraus SR, Brubaker L, Lin F, Richter HE, Bradley CS, Grady D (2006). The “costs” of urinary incontinence for women. Obstet Gynecol.

[16] Wall LL, Karshima JA, Kirschner CA, Arrowsmith SD (2004). The obstetric vesicovaginal fistula: characteristics of 899 patients from Jos. Nigeria Am J Obstet Gynecol.

[17] Jones A D: Living testimony: Obstetric Fistula and Inequities in Maternal Health. In*.* New York: UNFPA and Family Care International; 2007.

[18] Bangser M (2006). Obstetric fistula and stigma. Lancet.

[19] Mselle L, Moland K, Evjen-Olsen B, Mvungi A, Kohi T (2011). “I am nothing”: experiences of loss among women suffering from severe birth injuries in Tanzania. BMC Womens Health.

[20] Roush Karen M (2009). Social Implications of Obstetric Fistula: An Integrative Review. J Midwifery Womens Health.

[21] Creanga Andrerea A, Iliyasu Zubairu, Arinaitwe Loyce K. An evaluation of the United Nations Population Fund/Uganda's Obstetric fistula program. New York, USA: UNFPA Publication; 2008.

[22] Gjerde JL, Rortveit G, Muleta M, Blystad A (2013). Silently waiting to heal: experiences among women living with urinary incontinence in northwest Ethiopia. Int Urogynecol J.

[23] Bangser M, Mehta M, Singer J, Daly C, Kamugumya C, Mwangomale A. Childbirth experiences of women with obstetric fistula in Tanzania and Uganda and their implications for fistula program development. Int Urogynecol J. 2010;1–8. 10.1007/s00192-010-1236-8 20798927

[24] Roenneburg ML, Genadry R, Wheeless CR (2006). Repair of obstetric vesicovaginal fistulas in Africa. Am J Obstet Gynecol.

[25] Ahmed S, Holtz SA (2007). Social and economic consequences of obstetric fistula: Life changed forever?. International Journal of Gynecology & Obstetrics.

[26] Muleta M, Hamlin EC, Fantahun M, Kennedy RC, Tafesse B (2008). Health and social problems encountered by treated and untreated obstetric fistula patients in rural Ethiopia. J Obstet Gynaecol Can.

[27] Goffman E (1963). Stigma. Notes on the management of spoiled identity.

[28] Folkman S, LazarusR S (1980). Analysis of coping in middle aged community sample. J Health Soc Behav.

[29] Link BG, Phelan JC (2001). Conceptualizing stigma. Annu Rev Sociol.

[30] Mahajan AP, Sayles JN, Patel VA, Remien RH, Sawires SR, Ortiz DJ, Szekeres G, Coates TJ (2008). Stigma in the HIV/AIDS epidemic: a review of the literature and recommendations for the way forward. AIDS.

[31] Kleinman A, Wang WZ, Li SC, Cheng XM, Dai XY, Li KT, Kleinman J (1995). The social course of epilepsy: Chronic illness as social experience in interior China. Soc Sci Med.

[32] Yang LH, Kleinman A, Link BG, Phelanc JC, Lee S, Good B (2007). Culture and stigma: Adding moral experience to stigma theory. Soc Sci Med.

[33] Herek GM (2002). Thinking about AIDS and stigma: A psychologist’s perspective. J Law Med Ethics.

[34] Carver CS, Scheier MF, Weintraub JK (1989). Assessing coping strategies: A theoretically based approach. J Pers Soc Psychol.

[35] Green J, Thorogood N (eds.): Qualitative Methods for Health Reseach, Second edn. Great Britain: MPG Books Group; 2010

[36] Krueger RA, Casey MA (eds.): Focus Groups: A Practical Guide for Applied Research, 4th edn. Thousand Oaks, California, USA: SAGE Publications, inc; 2008

[37] Halloway I (2008). A-Z of qualitative research in health care.

[38] Dahlgren L, Emmelin M, Winkvist A (2004). Qualitative methodology for international public health.

[39] Kitzinger J (ed.): Focus group research: using group dynaemics to explore perceptions, experiences and understandings. London: Open University Press; 2005.

[40] Khan M, Manderson L (1992). Focus groups in Tropical diseases research. Health Policy Plan.

[41] Graneheim UH, Lundman B (2004). Qualitative content analysis in nursing research: concepts, procedures and measures to achieve trustworthiness. Nurse Educ Today.

[42] ICT Services and System Development and Division of Epidemiology and Global Health (2013). OpenCode 3.4. Umeå: Umeå University; 2013 [Available from: http://www.phmed.umu.se/english/units/epidemiology/research/open-code/ [cited 2015 September 5]]

[43] MOH: The National Policy Guidelines and Service Standards for Sexual and Reproductive Health and Rights. In*.* Edited by Reproductive Health Division DoCH, 3 edn. Kampala, Uganda: Ministry of Health.; 2012.

[44] Uganda National Council for Science and Technology: National Guidelines for Research involving Humans as Research Participants. In*.* Kampala -Uganda: UNCST; 2007.

[45] Yeakey MP, Chipeta E, Taulo F, Tsui AO. The lived experience of Malawian women with obstetric fistula. Culture, Health & Sexuality. 2009;11(5):499–13. 10.1080/13691050902874777 19444686

[46] Maulet N, Berthé A, Traoré S, Macq J. Obstetric Fistula "Disease" and Ensuing Care: Patients' Views in West-Africa. Afr J Reprod Health. 2015;19(1):112–23. 26103701

[47] Mwini-Nyaledzigbor PP, Agana AA, Beryl Pilkington F (2013). Lived Experiences of Ghanaian Women With Obstetric Fistula. Health Care Women Int.

[48] Okoye UO, Emma-Echiegu N, Tanyi PL (2014). Living with vesico-vaginal fistula: experiences of women awaiting repairs in Ebonyi State. Tanzan J Health Res.

[49] Weston K, Mutiso S, Mwangi Judy W, Qureshi Z, Beard J, Venkat P (2011). Depression among women with obstetric fistula in Kenya. Int J Gynecol Obstet.

[50] Yenenesh Tadesse G (2014). A qualitative study of the experience of obstetric fistula survivors in Addis Ababa, Ethiopia. Int J Women’s Health.

[51] Mehta M, Bangser M, Barber N, Lindsay J. Sharing the Burden: Ugandan Women Speak About Obstetric Fistula. Dar es Salaam, Tanzania: Women's Dignity Project and Engender Health; 2007.

[52] Wyrod R (2008). Between Women’s Rights and Men’s Authority: Masculinity and Shifting Discourses of Gender Difference in Urban Uganda. Gend. Soc.

[53] Khisa Anne M, Nyamongo Isaac K (2012). Still living with fistula: an exploratory study of the experience of women with obstetric fistula following corrective surgery in West Pokot. Reprod. Health Matters.

[54] Mehta M, Bangser M. Risks and resilience: Obstetric Fistula in Tanzania. Dar es Salaam: Women’s Dignity Project and Engender Health; 2006.

[55] Tebeu Pierre M, Maninzou Suzy D, Kengne Fosso G, Jemea B, Fomulu Joseph N, Rochat Charles H (2012). Risk factors for obstetric vesicovaginal fistula at University Teaching Hospital, Yaoundé, Cameroon. Int J Gynecol Obstet.

